# Supplementary material for: Is it easier to use one language variety at a time, or mix them? An investigation of voluntary language switching with bidialectals
Source: PLoS One. 2021 Sep 8;16(9):e0256554. doi: 10.1371/journal.pone.0256554 (PMC8425545; doi:10.1371/journal.pone.0256554)
Supplement: S1 Appendix — (DOCX) [file pone.0256554.s001.docx]

S1 Appendix

Proposed Standard English and Dundonian non-cognate stimuli.

| Standard English | Dundonian |
| --- | --- |
| Armpit | Oxter |
| Boy | Laddie |
| Cast | Stookie |
| Children | Bairns |
| Chimney | Lum |
| Ears | Lugs |
| Earwig | Forkytaily |
| Face | Pus |
| Girl | Lassie |
| Hill | Brae |
| Kerb | Cribby |
| Lake | Loch |
| Notebook | Jotter |
| Onion | Ingin |
| Potato | Tattie |
| Sandwich | Piece |
| Slippers | Baffies |
| Spot | Plook |
| Stream | Burn |
| Turnips | Neeps |
