# Supplementary material for: Is it easier to use one language variety at a time, or mix them? An investigation of voluntary language switching with bidialectals
Source: PLoS One. 2021 Sep 8;16(9):e0256554. doi: 10.1371/journal.pone.0256554 (PMC8425545; doi:10.1371/journal.pone.0256554)
Supplement: Attachment — Submitted filename: PONE-D-21-10597 Reviewer Comments.docx [file pone.0256554.s002.docx]

PONE-D-21-10596 Reviewer Comments

The proposed study seeks to determine if similar language control mechanisms are at play when bidialectals voluntarily switch between their two language varieties as when bilinguals switch between their two languages. Specifically, they seek to replicate the symmetric switch costs observed during voluntary language switching—in contrast to the asymmetric switch costs often observed in the cued language switching paradigm. In addition, they seek to replicate the mixing benefit also found during voluntary language switching, whereby naming on stay trials in a mixed-language block is overall faster than naming in single-language blocks. To accomplish this, the authors will have 46 bidialectal speakers of Scottish Standard English and Dundonian Scots perform a picture naming task consisting of two single-variety blocks and four voluntary mixed-variety blocks. Participants will name non-cognate pictures, and reaction times and accuracy will be analyzed to examine both switch costs and a global mixing benefit.

I believe that this study would make an excellent contribution to the field on language control. In fact, an emerging question that deserves more attention, and one that the proposed study addresses, is to what extent bilingualism and bidialectalism are different from one another, or if they exist along a continuum where the differences are quantitative (e.g., degree of overlap between the two languages/varieties) rather than qualitative. I am eager to see the results of this experiment and how they will contribute to this discussion.

The main points I would like the authors to address are summarized below, with detailed explanations following:

- What is the justification for the block order single-voluntary-single? Do the authors expect any spillover effects from the voluntary blocks on to the final single block? If so, how might they deal with this?
- Have the authors considered looking at performance over time as a function of trial number?
- How will the significance of fixed effects terms be determined?
- Will the authors examine any covariates in their analyses, such as including mean switch rate or self-rated measures from the questionnaire?
- Have the authors considered including a non-linguistic switching task, or a task that measures proactive/reactive control?

I have one main comment about the methodology that I would like the authors to either address or justify. The authors state that “participants will first see one single language block, followed by the four voluntary language switching blocks, and then again one single language block in the other language variety” (p. 9). What is the justification for placing the voluntary language switching block between the two single-language blocks, rather than placing the two single-language blocks at the beginning? I imagine it may be related to any switch costs that may emerge between the two single-language blocks, particularly when the non-dominant variety is named before the dominant variety. However, I’m also concerned about what effects the voluntary language mixing block might have on the following single-language block. For example, if the voluntary blocks disengage proactive control mechanisms, then this may persist into the following single-language block. If it does, then one might expect *worse* performance in this block, given that interference between the two varieties may be greater if proactive control is reduced (or, takes time to come back ‘online’). This would bias the results towards a mixing advantage, with slower/less accurate performance in the single-language blocks compared to the voluntary blocks, perhaps driven by any spillover effects from the voluntary blocks onto the following single-language block.

I am not sure there is a right answer on how to address this issue; switch costs between the two single-language blocks, if placed before (or even after) the voluntary blocks, would also bias the results towards a mixing advantage. Perhaps keeping the design as is, but placing the questionnaire after the voluntary blocks as a sort of ‘buffer’ before the final single-language block is an option? Please, let me know your reasoning for this particular order, and how you might address these in your results.

I also wonder to what extent the authors might be able to, at least partially, address this in their analyses. For example, it may be worthwhile to perform a time-series analyses looking at performance over time (e.g., across trials). I would recommend generalized additive mixed-effects models (GAMMs). It is possible in this framework to compare both the overall differences between blocks (in terms of their intercepts/overall height differences) as well as differences in how performance changes over time (by examining difference smooths). Some useful resources would be Sóskuthy (2021; *Journal of Phonetics, 84*), Wieling (2018, *Journal of Phonetics, 70*), and van Rij et al., (2019; *Trends in Hearing, 23*). All three authors have extensive R tutorials on their respective websites for performing time-series analyses with GAMMs.

In addition, I might also recommend the use of the buildmer (Voeten, 2021) package in R for the automatic simplification and testing of both random and fixed effects. The buildmer function builds on both lme4 and lmerTest and allows for a systematic and replicable way of simplifying models that fail to converge due to complicated random slopes. In addition, it is a nice compromise between Barr and colleagues’ “keep it maximal” approach and Bates and colleagues “keep it parsimonious” approach. The buildmer function starts with the maximally specified model, and attempts to fit it. In the event of a convergence failure, the function then automatically removes terms from the random slopes, determining the optimal combination that allows the model to converge. In other words, it automatically provides the maximal random slopes specifications that are possible. It’s a great time-saver, and it likewise makes replication easier for other researchers. Lastly, it also allows for the testing of fixed effects using functions from the lmerTest package. For continuous dependent variables (RTs), I recommend using Satterthwaite denominator degrees of freedom in the calculation of the p-values, while for binomial/logistic data (accuracy), Wald tests are the most optimal. It would also be useful, regardless of which method the authors use, to state how you will determine significance (e.g., based on t-values, by using omnibus tests, or by performing backwards/forwards term elimination/addition).

Also, have the authors considered including any covariates in their analyses of RTs and accuracy? For example, mean switch rate would be informative to look at, as would other measures from the questionnaire (e.g., any self-rated proficiency or usage measures). One might predict that those with greater proficiency in both varieties would show reduced costs overall, or perhaps a stronger mixing advantage.

Lastly, I recommend that the authors include a non-linguistic switching task or a measure of cognitive control, as these are both crucial to understanding what mechanisms are at play in the linguistic task. For example, the authors may use a number-letter task (e.g., Rogers & Monsell, 1995, *Journal of Experimental Psychology: General, 124*; Miyake et al., 2000, *Cognitive Psychology, 41*) as a measure of non-linguistic task-switching, and the AX-CPT (TS Braver and colleagues) as a measure of proactive and reactive cognitive control.
